# Overexpression of ezrin and galectin-3 as predictors of poor prognosis of cervical cancer

**DOI:** 10.1590/1414-431X20165356

**Published:** 2017-03-23

**Authors:** M. Li, Y.M. Feng, S.Q. Fang

**Affiliations:** Department of Obstetrics, The Second People's Hospital of Huaian, Huaian, Jiangsu Province, China

**Keywords:** Ezrin protein, Galectin-3 protein, Cervical cancer, Deep myometrial invasion, Lymph node metastasis, Prognosis

## Abstract

The aim of this study was to explore the correlation of ezrin and galectin-3 expressions with prognosis in cervical cancer. The immunohistochemical method was applied to detect ezrin and galectin-3 expressions in normal cervix tissues (n=30), cervicitis tissues (n=28), cervical intraepithelial neoplasia (CIN) tissues (classified as I-III, n=89), and cervical carcinoma tissues (n=84). Follow-up was conducted for 5 to 78 months to analyze the correlation of protein expressions with prognosis. Ezrin and galectin-3 expressions in cervical cancer were significantly higher than in normal cervix, cervicitis and CIN (all P<0.05), and expressions in CIN were significantly higher than in normal cervix and cervicitis (both P<0.05). The expressions of ezrin and galectin-3 were both related with histological grade, deep myometrial invasion and lymph node metastasis (all P<0.05). Spearman analysis showed that ezrin expression was positively correlated with galectin-3 expression in cervical cancer (r=0.355, P<0.05). The survival rate of patients with high expressions of ezrin and galectin-3 was significantly lower than those with low expressions of proteins (both P<0.05). The expressions of ezrin and galectin-3, histological grade, depth of stromal invasion, and lymph node metastasis are risk factors affecting the survival rate of patients with cervical cancer. The expressions of ezrin and galectin-3 were correlated with the development of cervical cancer, and overexpressions of those proteins were indicative of poor prognosis in patients with cervical cancer.

## Introduction

Cervical cancer is one of the most common gynecologic cancers in women worldwide, second to breast cancer, accounting for around 13% of both total cancer cases and total cancer deaths in women (1). Studies have shown that there are more than 500,000 patients with cervical cancer, of which almost 80% are in the developing countries (2,3). A low 5-year survival rate was reported for patients with cervical cancer, especially in Indians, who are responsible for 26.5% of the global burden, and cervical cancer shows a decreasing trend in the onset age worldwide (4). Cervical cancer is difficult to diagnose due to subtle and nonspecific initial symptoms in early stage. However, vaginal bleeding, vaginal discharge, abdominal pain and other symptoms may occur as the disease progresses (4). The etiology factors for cervical cancer include: smoking, sexual factors, reproductive factors, nutritional factors (like lack of folic acid) and genetic factors (5). At present, surgery and radiotherapy are the two commonly used treatment methods for cervical cancer, however, treatment for patients with advanced cervical cancer is not satisfactory. With the development of molecular biology, increasing research is attempting to find new effective targets in biomolecular aspects (6,7).

Ezrin, a membrane-cytoskeletal linking protein, mediates the connection of the membrane to cytoskeleton. Therefore, ezrin plays an important role in cell mitosis, movement, migration and other physiological functions (8). Studies have shown that the invasion of ezrin protein is related to metastasis of tumor cells, such as from osteosarcoma and lung cancer, and is considered a key regulator of metastasis (9,10). In addition, it has been previously indicated that the aberrant localization and overexpression of ezrin could be an independent effective biomarker for prognostic evaluation of early stage cervical cancer (8). Galectin-3 belongs to the lectin family, a galactosidase binding protein with chimera structure. Not only it is important for interactions between cells and cell matrix, cell growth, cell cycle regulation, apoptosis, cell injury and repair, but it is closely related to the proliferation, tumor transformation and metastasis process (11,12). Several studies disclosed that the over-expression of galectin-3 is closely related to the development of many human tumors, such as large-cell lymphoma, colorectal cancer, breast cancer, liver cancer, brain tumors, melanoma and thyroid cancer (13,14). The role of galectin-3 in vascular endothelial growth factor C (VEGF-C)-induced cervical cancer cell invasion has been previously investigated, and silencing of galectin-3 expression with specific siRNA largely impaired VEGF-C-enhanced invasion in cervical carcinoma cell line (15). Currently, few studies have focused on the expression of ezrin and galectin-3 protein in cervical cancer. This paper will demonstrate the relationship between ezrin and galectin-3 expressions and the development of cervical cancer and its prognosis by detecting their expressions in cervical, normal cervix, cervicitis, and cervical intraepithelial neoplasia (CIN) tissues.

## Material and Methods

### Study subjects

Between May 2007 and April 2009, cervical tissue samples from 201 women with cervical disease admitted in the Second People's Hospital of Huaian were collected. Among them there were 28 cases with cervicitis, 89 cases with CIN (classified as I–III) and 84 cases of cervical cancer (36 cases of Stage I–II, 48 cases of Stage III–IV); there were 63 cases of squamous cell carcinoma, 13 cases of adenocarcinoma, and 8 cases of adeno-squamous carcinoma according to the histological classification (16). CIN grade (17) was described as follows: CIN grade I is mild dysplasia, with minor cell atypia arranged irregularly, but polarity still maintained, and abnormal proliferation of cells confined to the basal 1/3 of the epithelium; CIN II is moderate dysplasia, with remarkable cell atypia arranged irregularly, and abnormal proliferation of cells occupying the basal 2/3 of the epithelium; CIN III is severe dysplasia, a significant epithelial cell atypia of severe dysplasia with polarity loss, also abnormal proliferation of epithelial cell spanning more than 2/3 or the whole epithelium. Clinical stages of cervical cancer are classified by International Federation of Gynecology and Obstetrics (FIGO) (18) as follows: Stage I, cervical cancer is confined to the cervix (the diffusion part to the Palace ignored); Stage II, the tumor grew beyond the uterus, but not in the pelvic wall or lower 1/3 of the vagina; Stage III, the tumor is extended to the pelvic wall and/or lower 1/3 of the vagina and/or cause renal pelvis or kidney dysfunction; Stage IV, the tumor invades the bladder mucosa and/or rectal mucosa and/or beyond the true pelvis. All patients with cervical disease were included in the study based on the following criteria: 1) Patients over 18 years of age with good compliance with treatment and observation; 2) Patients diagnosed by a pathology slice reviewed by a pathologist; 3) medical records maintained intact, with all cases for the initial onset and no history of receiving radiotherapy, chemotherapy and biological therapy; 4) Patients without previous history of malignancy. Exclusion criteria for patients with cervical disease were: 1) Congenital, hereditary, autoimmune and cardiovascular diseases; 2) Other diseases within the uterus except cervix uterus; 3) Patients in pregnancy or breast-feeding; 4) Patients with serious history of smoking, alcoholism, drug abuse or mental illness. Normal cervical tissue samples of 30 uterine fibroids cases were taken by hysterectomy. Cervical cancer patients were followed-up for 5 to 78 months by telephone interviews and routine return visits to the clinic with the average duration of 58.4 months. At the end of the follow-up, 19 patients died and 17 were lost to follow-up. This study was approved by the Hospital Ethics Committee with signed informed consent obtained from each patient.

### Immunohistochemical method

The samples were dehydrated using graded ethanol (S27074102, Sinopharm Chemical Reagent Co., Ltd., China), fixed with dimethylbenzene (10023418, Sinopharm Chemical Reagent Co., Ltd.), and embedded in paraffin (SA633001, Sinopharm Chemical Reagent Co., Ltd.), followed by sectioning into slices (Polysine Adhesion Slides; SLI-2002, Fuzhou Maixin Biotech. Co., Ltd., China). Subsequently, the sections were dewaxed with xylene. Then the samples were dehydrated using graded ethanol, and washed with PBS. Antigen retrieval was conducted using a pressure cooker: paraffin sections were put into the pressure cooker and pressurization was increased slowly. After the pressure cooker emitted steam for 5-6 min, the paraffin sections were removed from the heat source and put into the cold water. Then, 1 drop of peroxidase (Beijing Zhongshan Golden Bridge Biotech. Co., Ltd., China) was added to each slice to block the reaction. After, the section was incubated at room temperature for 10 min, washed with PBS, and normal non-immune serum was added and slides were incubated for 10 min. Subsequently, the antibodies were added (mouse anti-human ezrin (ab4069, 1:100), galectin-3 monoclonal antibody (ab2785, 1:100), PBS as blank control) (All purchased from Santa Cruz Company, USA) and incubated overnight and washed with PBS. Then, biotin-labeled secondary antibody was added (69314360, Sinopharm Chemical Reagent Co., Ltd.) and incubated for 10 min, followed by washing with PBS. Additionally, streptavidin enzyme was added and samples were incubated for 10 min. Again, samples were washed with PBS, and added with DAB solution (SP-9000-D, Beijing Zhongshan Golden Bridge Biotech. Co., Ltd.). After washing with PBS, the samples were hematoxylin stained (CTS-1097, Fuzhou Maixin Biotech. Co.), colored in blue and dehydrated with gradient ethanol. Xylene was used for transparency and neutral gum (DAB-0033, Fuzhou Maixin Biotech. Co.) was used for sealing.

### Evaluation of immunohistochemical method

Galectin-3 protein is mainly distributed in the cytosol, expressed in the nucleus and the cell surface, and positive results are assessed by the appearance of brown particles in the cytoplasm and/or nucleus. Ezrin protein mainly exists in the cytoplasm, some in the cell membrane, and positive results are assessed by visible brown particles in cell membranes and cytoplasm without nuclear membrane staining. At high magnification using a V-130B10C model (Shenzhen Boshida Optical Instrument Co., China), 5 photos were taken in each corner and central portion of the slice, and the proportion of positive cells in each 100 cancer cells was calculated, and averaged. The results for different portions of positive cells were divided into four grades: <10%: 0 point; 10–40%: 1 point; 41–70%: 2 points; >70%: 3 points. These results were divided into four grades by graduation of stained cells color: 0 for no color; 1 point for pale yellow; 2 points for pale brown; 3 points for brown. These two values were categorized into: 0–3 points as (–): 4–6 points as (+); 7–9 points as (++); 10–12 as (+++). For statistical analysis, these categories were dichotomized in negative (–), and positive (remaining categories) (19).

### Statistical analysis

SPSS 20.0 statistical software (USA) was applied for statistical analysis. Data are reported as means±SD. The *t*-test was used for comparison between the two groups, and one-way analysis of variance was used among multiple groups. Count data are reported as a percentage or ratio. The chi-square test was used between two groups, and the rank-sum test was used for ranked data. Correlation analysis was performed using Spearman rank correlation analysis. Survival is reported as Kaplan-Meier curves. Log-rank test was used for comparison between two groups, and Cox regression model was applied in multivariate analysis. P<0.05 was considered to be statistically significant.

## Results

The differences in average age of patients in each group, the number of people <40 and ≥40 years of age, and body mass index (BMI) were not statistically significant (all P>0.05). In the 84 cases of cervical cancer, there were 20 well-differentiated, 29 moderately differentiated and 35 poorly differentiated cases. There were 36 cases in stages I+II, 48 cases in stages III+IV. There were 56 cases with tumor diameter ≤4cm and 28 cases with tumor diameter >4cm. There were 39 cases with ≤1/2 interstitial infiltration and 45 cases >1/2, and 55 cases with lymph node metastasis and 29 cases without lymph node metastasis (Table 1).

**Table 1:**
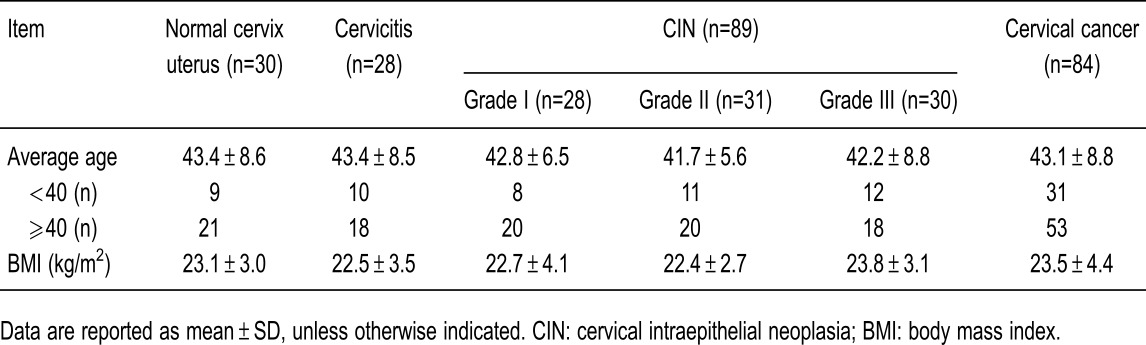
Baseline characteristics in each group.

### Comparison of ezrin and galectin-3 expressions

**Figure 1:**
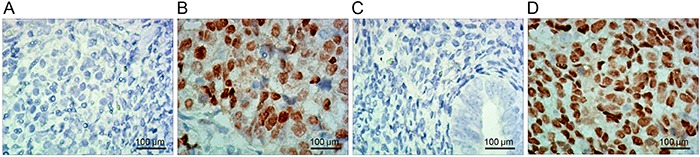
Protein expression of ezrin and galectin-3 in each group. A, Negative ezrin protein expression in cervicitis tissue; B, Positive ezrin protein expression in cervical cancer group; C, Negative galectin-3 protein expression in cervicitis group; D, Positive galectin-3 protein expression in cervical cancer group.

The positive ezrin and galectin-3 protein expression indicated that cytoplasm was stained brown (Figure 1 and Table 2). The positive protein expression rates in CIN, and CIN grade I, II, and III and in cervical cancer were significantly higher than normal cervix group (all P<0.05). The positive expressions of ezrin and galectin-3 in CIN group were higher than in cervicitis group (both P<0.05), but the differences of positive ezrin expression rate among CIN grade I, II and III were not statistically significant (all P*>*0.05). By comparison, the positive galectin-3 expression rate improved with grade elevation of CIN (from I to III), and the difference was statistically significant (all P<0.05). The positive expression rates of ezrin and galectin-3 protein in cervical cancer were significantly higher than cervicitis group, and CIN grade I, II, and III groups (all P<0.05).

**Table 2:**
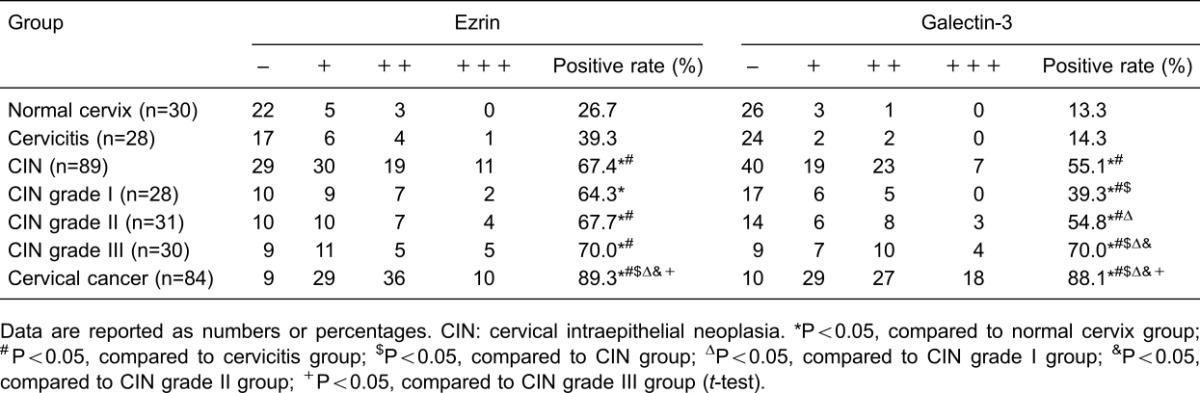
Positive expression rate of ezrin and galectin-3 proteins in each group.

### Relationship of clinical pathological features of cervical cancer with the expressions of ezrin and galectin-3 proteins

Table 3 shows that the protein expressions of ezrin and galectin-3 were related to histological grade, FIGO stage, stromal invasion and lymph node metastasis (all P<0.05). The positive ezrin and galectin-3 protein expressions in poorly differentiated patients were significantly higher than in the well-differentiated patients (all P<0.05), but were not correlated with age, menopausal status, histological type or tumor size (all P>0.05).

**Table 3:**
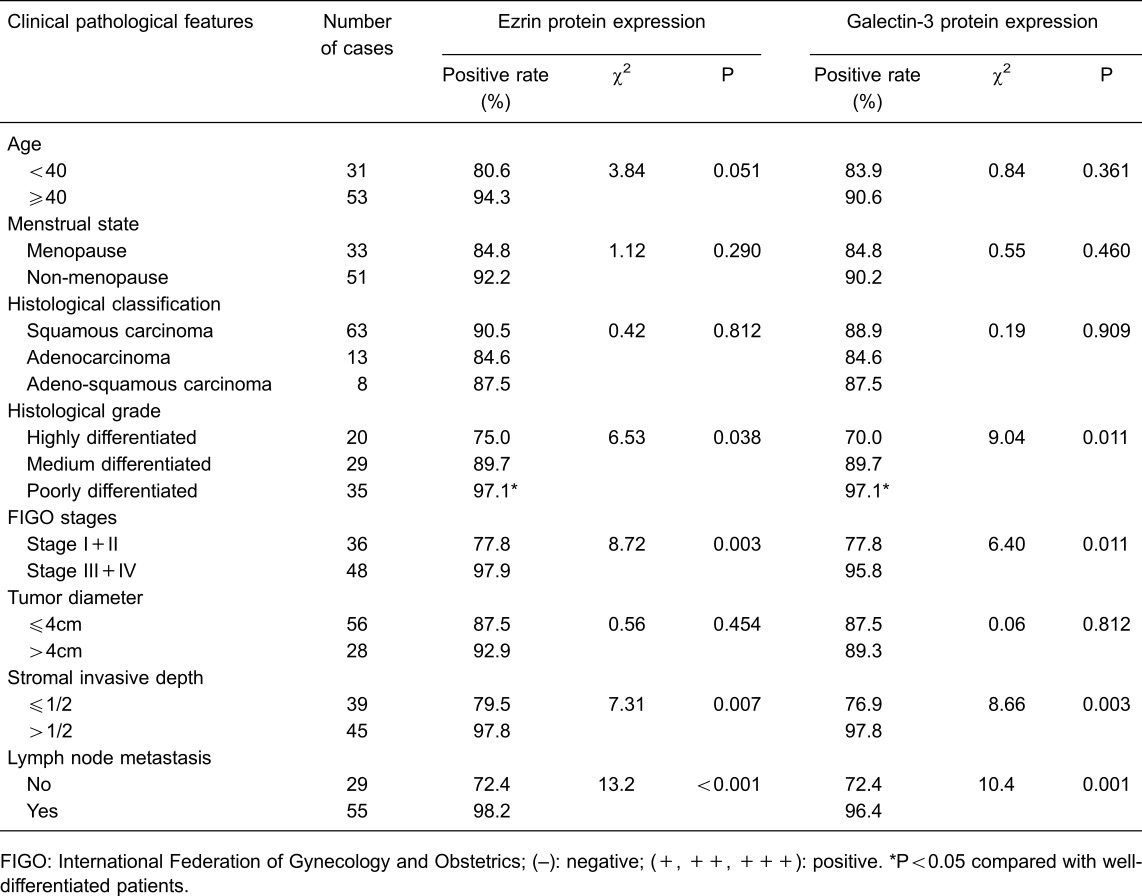
Relationship of ezrin and galectin-3 protein expression with clinical pathological features of cervical cancer.

### Correlations of ezrin expression with galectin-3 expression in cervical cancer

A significant correlation between ezrin and galectin-3 protein expressions in cervical cancer was found and is shown in Table 4 (r=0.355, P<0.05).

**Table 4:**
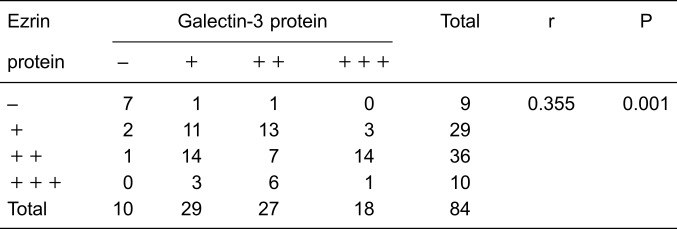
Correlations between ezrin and galectin-3 protein expression in cervical cancer.

### Relationship of ezrin and galectin-3 expressions with cervical cancer prognosis

**Figure 2:**
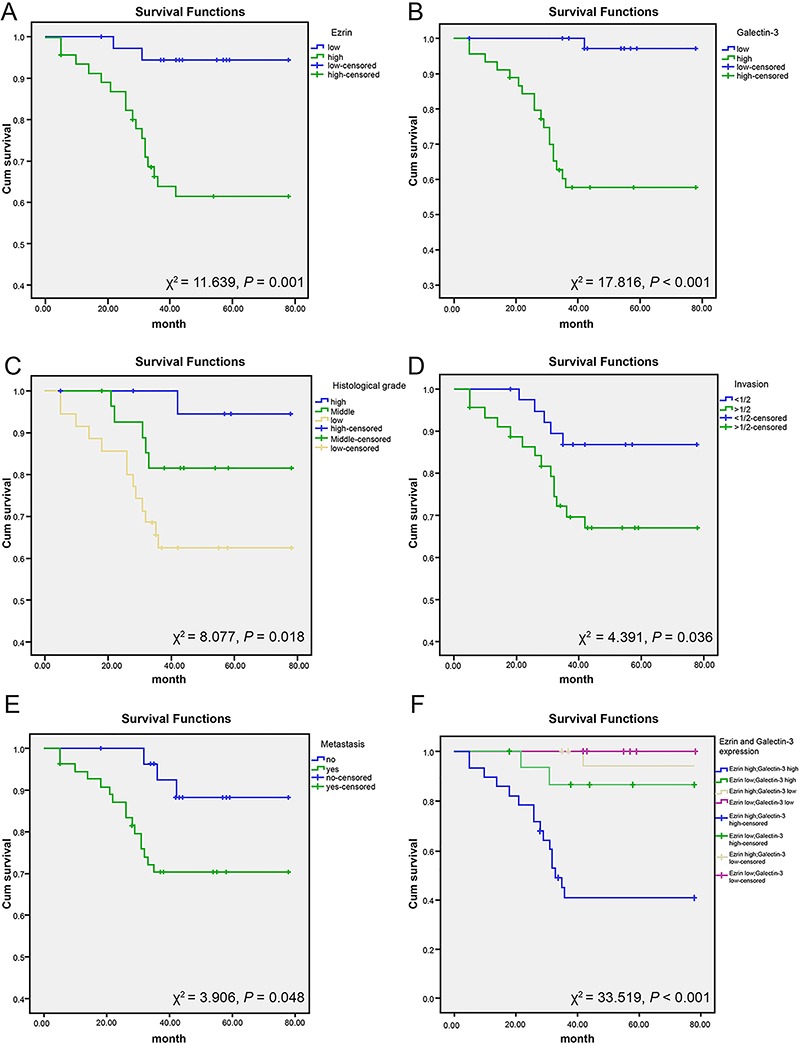
Kaplan-Meier curves for ezrin (*A*), galectin-3 (*B*), histological grade (*C*), stromal invasive depth (*D*), lymph node metastasis (*E*), and overexpression of ezrin and galectin-3 (*F*). cum survival: cumulative survival.

Patients with cervical cancer were followed-up from 5 to 78 months. Ezrin and galectin-3 expressions in cervical cancer scored – and + were grouped into low expression group, while ++ and +++ were grouped into high expression group. Kaplan-Meier survival curves of ezrin and galectin-3 protein expression with their influential factors including histological grade, stromal invasive depth and lymph node metastasis factors are shown in Figure 2. The survival rate for cervical cancer patients with high expressions of ezrin and galectin-3 was significantly lower than those with low expressions of the proteins (both P<0.05). The survival rate was lower with low histological grade compared to high, and the difference between well differentiated group and medium differentiated group was also statistically significant (P<0.05). Survival rate of patients with superficial muscle invasion was significantly higher than those with deep myometrial invasion (P<0.05). Survival rate with no lymph node metastasis was significantly higher than with lymph node metastasis (P<0.05). The survival rates of patients with high expression of ezrin+galectin-3 were significantly lower than those with low expression of ezrin + high expression of galectin-3, and those with high expression of ezrin + low expression of galectin-3, and those with low expression of ezrin + galectin-3 simultaneously (all P<0.05). With the inclusion of influential factors (P<0.05) of Kaplan-Meier analysis into the results of COX proportional hazards model analysis, it was found that ezrin and galectin-3 protein expression, histological grade, stromal invasive depth and lymph node metastasis were risk factors for cervical cancer prognosis (Table 5).

**Table 5:**
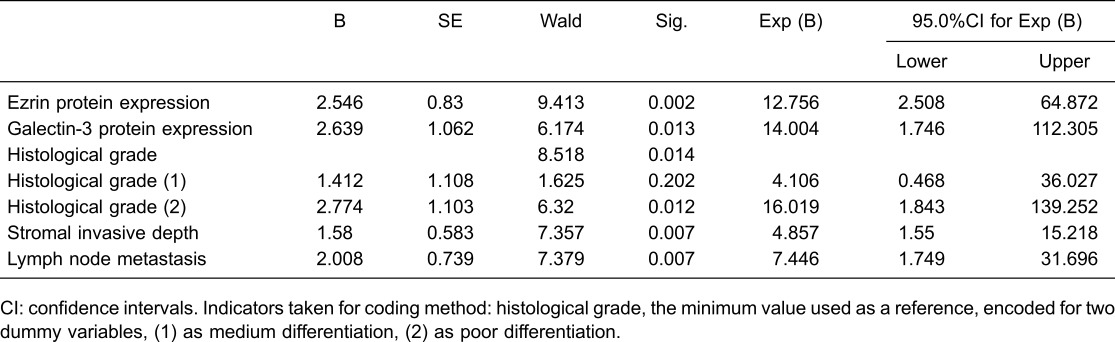
Multivariate COX regression analysis of patients with cervical cancer

## Discussion

Cervical cancer is regarded as an epidemic, usually caused by infection with specific types of human papillomavirus (HPV) (20). Although mortality rates have decreased significantly with advanced treatment, poor therapeutic effect still exist for most patients, as well as poor prognosis. The identification of important substances in cervical cancer may contribute to a comprehensive understanding of the oncogenic mechanism, therefore, providing indicators for prognostic tests.

The study found that ezrin expression was related to the development and prognosis of cervical cancer. Ezrin belongs to ERM protein family, and the gene is located in 6q25, composed of 585 amino acids with certain specificity (21). Ezrin protein can reduce expression of E-cadherin on the cell surface, thereby reducing the adhesion between cells, resulting in the migration to a distant place. In addition, ezrin protein may also gather in CD44 cytoplasmic region, which would cause the cytoskeleton reconstruction with distant metastasis of cells. The overexpression of ezrin protein would promote the worsening of tumors (22). Several studies disclosed that ezrin proteins play an important role in the development of cancer (23). For example, in patients with osteosarcoma, ezrin protein expression level *in vivo* is higher than normal. While the expression of ezrin protein is decreased by siRNA, the proliferation, invasion and migration of osteosarcoma cells were significantly inhibited, which is indicative of the essential function of ezrin overexpression in those processes (24). Tan et al. analyzed the protein expression level in 56 cervical cancer cases, and the results showed that the amount of ezrin expression in cervical tissue was related with tumor progression (23). In addition, Kong et al. (8) reported overexpressed ezrin in cervical cancer, closely related with poor differentiation, late stage, and lymph node metastasis, as well as poorer 10-year survival rate for patients with early stage cervical cancer. Further, ezrin was implicated as an EMT regulator and tumor promoter in cervical cancer, and downregulation of ezrin suppressed cervical cancer progression, possibly via the phosphoinositide 3-kinase/Akt pathway (25).

This study also found that galectin-3 protein expression was related with the development and prognosis of cervical cancer. Galectin-3 gene is located in chromosome lpl3 and 14q21-22, with its relative molecular mass of 26152 Da. It has three structurally distinct regions on galectin-3 protein: one containing a short NH_2_-terminal of 12 amino acids, and control cell target function. One structure is mainly rich in glycine, proline, tyrosine and collagen-like substances and relevant with combination of cell surface decorated with glycoprotein complex, which can also be used as matrix metalloproteinase substrate. The other structure is in carboxyl-terminal region, an iconic structure of galectin-3, containing functional sugar-binding domain, which can identify β-galactose residue-heterosexual and bind with it (26). Under normal circumstances, galectin-3 protein can be expressed in many tissues, and research shows that it would overexpress constantly due to the severity of the disease in a variety of tumor tissues (26). With the effect on cell surface molecules, extracellular matrix proteins, and glycoproteins within the cell, it participates in cell proliferation, apoptosis, adhesion, angiogenesis, splicing of precursor messenger RNA, etc. It also plays an important role in the development and metastasis of tumors (27,28). Povegliano et al. (29) discovered that galectin-3 protein is highly expressed in colorectal cancer tumor tissues, and in tissues with the disease development or recurrence its expression was significantly increased. It has also been shown that galectin-3 is detected in gastric adenocarcinoma, colorectal cancer and other cancers (30,31). Some scientists thought that galectin-3 within the nucleus regulates Wnt/β-catenin signaling pathway mainly by activation of the transcription of *CyclinD1*, *C-myc* and other genes, to enhance the expression of its target genes, leading to tumorigenesis and adverse effect on prognosis (32). The function of galectin-3 in promoting cell survival and potentially inducing chemo-resistance and T cell apoptosis might explain the correlation between galectin-3 overexpression and poor prognosis (33–35). Additionally, the phenotype of cells expressing galectin-1, -3 and -9 and the association with clinico-pathological parameters in cervical cancer has been previously investigated. Galectin-3 was suggested to be expressed by tumor cells in 84% of samples, and it might have dual functions: weak expression correlated with increased tumor invasion and growth, while positive expression with decreased invasion and growth (36). Inconsistent with our results, galectin-3 expression was reported to be down-regulated in cervical cancer tissues and the decreased expression is associated with the progression of cervical neoplasia (37). In addition, inconsistent results have been described for galectin-3 concerning prognosis in different tumor types. Its prognostic or diagnostic value in cervical cancer need to be further confirmed due to diverse functions and correlations with clinico-pathological parameters and survival (38–40).

In conclusion, this study showed that the expressions of ezrin and galectin-3 protein may be associated with the development of cervical cancer and their overexpressions may indicate a poor prognosis of this disease. The mechanisms of ezrin and galectin-3 in the process of development and prognosis for cervical cancer remains to be elucidated, and further research is required.
